# Efficient Network Architecture Search Using Hybrid Optimizer

**DOI:** 10.3390/e24050656

**Published:** 2022-05-06

**Authors:** Ting-Ting Wang, Shu-Chuan Chu, Chia-Cheng Hu, Han-Dong Jia, Jeng-Shyang Pan

**Affiliations:** 1 College of Computer Science and Engineering, Shandong University of Science and Technology, Qingdao 266590, China; 202082060058@sdust.edu.cn (T.-T.W.); hdjia@sdust.edu.cn (H.-D.J.); jspan@cc.kuas.edu.tw (J.-S.P.); 2 College of Artificial Intelligence, Yango University, Fuzhou 350015, China; cchu@cna.edu.tw; 3 Department of Information Management, Chaoyang University of Technology, Taichung 41349, Taiwan

**Keywords:** convolutional neural network, Aquila optimization, residual block, neural architecture search

## Abstract

Manually designing a convolutional neural network (CNN) is an important deep learning method for solving the problem of image classification. However, most of the existing CNN structure designs consume a significant amount of time and computing resources. Over the years, the demand for neural architecture search (NAS) methods has been on the rise. Therefore, we propose a novel deep architecture generation model based on Aquila optimization (AO) and a genetic algorithm (GA). The main contributions of this paper are as follows: Firstly, a new encoding strategy representing the CNN coding structure is proposed, so that the evolutionary computing algorithm can be combined with CNN. Secondly, a new mechanism for updating location is proposed, which incorporates three typical operators from GA cleverly into the model we have designed so that the model can find the optimal solution in the limited search space. Thirdly, the proposed method can deal with the variable-length CNN structure by adding skip connections. Fourthly, combining traditional CNN layers and residual blocks and introducing a grouping strategy provides greater possibilities for searching for the optimal CNN structure. Additionally, we use two notable datasets, consisting of the MNIST and CIFAR-10 datasets for model evaluation. The experimental results show that our proposed model has good results in terms of search accuracy and time.

## 1. Introduction

CNN is a widely used neural network architecture which is formulated by biologists Huber and Wiesel in their early work on cat visual cortices. It can be applied to many problems in image classification and segmentation [1,2,3] and natural language processing (NLP) [4,5]. However, designing CNN structures completely manually is a complex task. For example, traditional network designs, such as LeNet [6], AlexNet [7], and ResNet [8]. These networks not only rely heavily on existing deep learning architectures when designing manually but also require the re-selection of appropriate structures and hyperparameters for specific tasks.

However, owing to the high-dimensional properties of the neural networks, neuroevolution is mainly employed for reinforcement learning of shallow networks. Stanley et al. attempted to address this question with hypercubic-enhanced topological neuroevolution (HyperNEAT) [9], which acts as an indirect coding method that can generate millions of networks, but still has a low fitness for optimal CNNs and is therefore considered more suitable as a feature extractor.

In 2016, some academics proposed an approach that would allow the network structure to be constructed not by humans, but by giving the machine some basic elements of a neural network and allowing it to automatically construct a better network. This approach is called neural architecture search (NAS) [10,11], which combines and selects the basic elements of a neural network by taking them into a limited search space and finally arriving at a network with good network performance. However, it is necessary to constantly try and combine different architectures in the process, which consumes a lot of time and requires a powerful amount of computation.

In perceptual tasks, the success of deep learning is closely connected to the automation of the feature extraction process, where hierarchical feature extractors automatically acquire the information that we need in an end-to-end manner. However, as we manually design increasingly complex neural architectures for specific tasks, our demand for architecture engineering is growing. NAS enables automated neural network architecture design and thus is the next step in the automation of machine learning. NAS can be considered as a subdomain of AutoML. Most modern deep neural network architectures are created based on human experience, and most of them require a long and tedious iterative experimentation process, while NAS enables the automatic generation of effective deep learning frameworks for specific tasks without any human intervention.

There are many methods to perform an automatic search by combining reinforcement learning, intelligent optimization algorithms, and gradient descent with CNNs. Baker et al. suggest a reinforcement learning (RL)-based metamodeling approach for automatically designing CNN architectures, called MetaQNN [12], by training an agent to sequentially determine the types of layers and their parameters. NASNet [13] uses validation accuracy as a reward signal, a recursive network of controllers optimized by policy gradients to generate the best neural structure description. EvoCNN [14] combines with the design of CNNs, which use GA for searching CNN structures with particular operations. IPPSO [15] is the first method to use particle swarm optimization (PSO) [16,17,18] in the design of CNN networks. It is inspired by the IP address setting, where each layer of the CNN is assigned an IP address. By the above setting, a new coding method is set. In addition, it uses PSO to automatically search for the optimal structure of the CNN at variable lengths without any human intervention in the process. However, this algorithm has limitations on particle length, and cannot be encoded at variable lengths, and the results are limited to three data sets. Based on the IPPSO drawbacks, psoCNN [19] proposes a new difference and velocity operator, respectively, and uses variable-length particles to explore the optimal solution of the CNN architecture. However, the main drawback is that the search space is only a combination of existing schemes and does not make maximum use of the solution space. Therefore, Lawrence et al. [20] use the group operation in psoCNN to maximize the search solution space and reduce the computational cost to a certain extent, but it is limited by the length of the particle size.

With deeper research in NAS, some studies are increasingly proved to be not perfect. Therefore, some more mature and complete solutions have been proposed in related works. ENAS [21] proposes a faster and more efficient search network structure using weight sharing, by which the time to obtain the optimal subnetwork is reduced. However, this approach may lead to weight shifts. DNS [22] reduces the drawbacks coming from ENAS by modularizing the search space. Although these above algorithms obtain better results on the corresponding data sets respectively, on the one hand, the time and space cost is relatively high, and on the other hand, there is no good way to deal with the continuous space search problem, and our algorithm solves these two problems relatively well.

Based on two metrics, classification accuracy and search speed, and finding the balance between the two metrics, We use the AO and GA to maximize the exploration of the CNN optimal structure, which we call HAGCNN. The primary contributions of this paper are as follows:

1. We design a new binary-based particle-encoding strategy that can efficiently encode CNN structures and develop new evolutionary computation algorithms based on the new encoding strategy;
2. A new approach to position updating is proposed, which focuses on combining the AO with GA. Crossover, Mutation, and Selection operations are added before the position update, thus allowing various directions and scales to search the solution space and increase the chances of diverse network formation;
3. The inclusion of skip connections allows the algorithm to search for reasonable CNN architectures at variable lengths and generate new models, which break the constraint of the fixed-length encoding of traditional AO;
4. To obtain better network performance, we introduce the residual block structure and group strategy, which enables us to explore deeper network structures sensibly.

The remainder of this paper is as follows: Section 2 provides a detailed description of the AO, GA, CNN, and residual block. Section 3 describes the proposed HAGCNN algorithm in detail. In Section 4, we introduce the experimental details we have performed. Section 5, we conclude and analyze the experimental results. Section 6 summarizes the whole paper and presents future works.

## 2. Background

### 2.1. CNN Introduction

**Figure 1:**
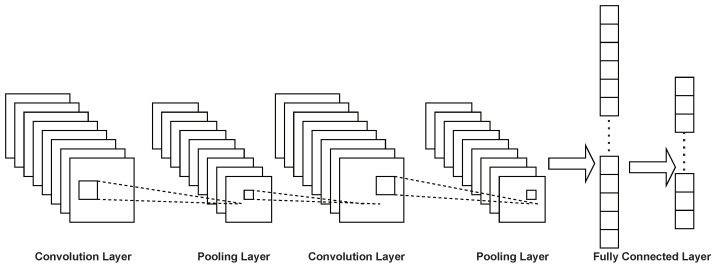
Normal CNN structure.

CNN [23,24,25,26] was proposed in the 1960s and discovered by Hubel and Wiesel while studying neurons in the cat brain cortex for local sensitivity and an orientation selection. CNN does not require to be manually designed during the whole-feature extraction but is processed by convolution operation directly. Deep convolutional neural networks mainly contain input layers, pooling layers, fully connected layers, convolutional layers, and an output layer. The input layer is used to receive the input data and the convolution layer is mainly used to perform feature extraction on the data. The data downsampling process is performed on the pooling layer. The fully connected layer is a deep network with the multi-Layer perceptron (MLP) [27,28,29]. Furthermore, it is mainly implemented through the activation function to the final output layer. The structure of CNN is shown in Figure 1. Deep convolutional neural networks stack various layers on top of each other and the output of each layer is calculated as the sum of the product of the input of each layer and the internal weights, all of which will be used as input to the next layer, resulting in a recognition classification result.

In training a CNN, tens of thousands of parameters may have to be processed because of the different architectures. It may take hours or even days, so it is important to develop an automatic search mechanism.

### 2.2. Residual Block

ResNet [8] is made up of a series of residual blocks, and a residual block can be expressed by Equation (1).

(1) $$X_{l+1}=X_{l}+F\left(X_{l},W_{l}\right)$$

The residual block is classified into two parts, which are the direct mapping and the residual section. Among them, $F(X_{l},W_{l})$ is the residual section that consists of two to three convolution operations.

In a convolutional network, $X_{l}$ may not have the same number of feature maps as $X_{l+1}$, and it is then necessary to use the convolution of 1×1 for dimensioning up or down, in which case the residual block representation is given in Equation (2).

(2) $$X_{l+1}=h\left(X_{l}\right)+F\left(X_{l},W_{l}\right)$$

where, $h\left(X_{l}\right)=W_{l}^{{}^{\prime }}X$, and $W_{l}^{{}^{\prime }}$ is the 1×1 convolution operation. However, generally this is only used when lifting the dimension.

### 2.3. Genetic Algorithm (GA)

The genetic algorithm (GA) [30,31] is a population-based optimization search algorithm derived from natural selection. It uses the concept of survival of the fittest. It tries to exploit the most suitable individuals through iteration, and it abstracts the problem space as a population of individuals. GA converts the original group of individuals to a group of individuals where each individual stands for the solution of the problem waiting to be solved. The computation is achieved by cycling through individuals in a population using crossover, variation, and selection.

To determine whether a particular string will be involved in the reproduction process, a selection operation is proposed. Here, there are many ways to select the best chromosome, for example roulette wheel selection, rank selection, tournament selection, Boltzmann selection, steady-state selection, and elitist selection, etc.

The crossover operation is implemented mainly by combining genetic information from two or more parents to generate offspring. This includes signal point, uniform, partial matching, two-point and k-point orders, crossover with priority retention, reduction of substitution, shuffling, and cycling.

The mutation operation is used to maintain genetic diversity from one population to the next. The well-known mutation operations are simple inversions, substitutions, and hybrid mutations.

### 2.4. Aquila Optimizer (AO)

The Aquila optimizer (AO) [32,33] is a population-based optimizer proposed in 2021. It is inspired by Aquila’s social behavior of catching prey. Similar to other population-based metaheuristics, the algorithm begins with *N* individuals and the initial population is *X*. We perform this initialization process in Equation (3):

(3) $$X_{ij}=rand\times \left(UB_{j}-LB_{j}\right)+LB_{j},i=1,2,\cdots ,N,j=1,2,3,\cdots ,Dim$$

where $UB_{j}$ and $LB_{j}$ are the upper and lower bounds of the *j*-th exploration space, rand is a randomly generated parameter in the range from 0 to 1, and Dim is the whole search space dimension.

The algorithm is divided into two processes: exploration and exploitation, in which there are four main implementation strategies. The algorithm can determine the possibilities of transferring the exploration stage to the exploitation stage by judging the condition $t\leq (\frac{2}{3})\times T$. where *t* and *T* stand for the current iteration and the maximum number of iterations, respectively.

In the first strategy, the Aquila discriminates the prey region and selects the best hunting area by a vertical swoop of high-altitude soaring. This strategy considers the average agent $X_{M}$ and the best agent $X_{b}$ in the process of exploration. The mathematical equation is as Equation (4):

(4) $$X_{i}\left(t+1\right)=X_{b}\left(t\right)\times \left(\frac{1-t}{T}\right)+\left(X_{M}\left(t\right)-X_{b}\left(t\right)\times rand\right)$$

(5) $$X_{M}\left(t\right)=\frac{1}{N}\sum_{i=1}^{N}X\left(t\right),\forall j=1,2,\cdots ,Dim$$

where $X_{i}\left(t+1\right)$, $X_{b}\left(t\right)$ denote the position of the individual at the (t+1)-th iteration number and the best solved individual obtained at the *t*-th iteration, respectively. $X_{M}\left(t\right)$ shows the average value of the current solution position at the *t*-th iteration, which is calculated by Equation (5). *t* and *T* indicate the current and maximum number of iterations, and rand is a random value between 0 and 1. Dim is the dimension size and *N* is the number of candidate solution.

In the second strategy, the individual position is updated on the basis of the L$\overset{´}{e}$vy flight $L\overset{´}{e}vy\left(D\right)$ distribution and $X_{b}$. A mathematical formulation of this strategy is given by Equation (6)

(6) $$X_{i}\left(t+1\right)=X_{b}\left(t\right)\times L\overset{´}{e}vy\left(D\right)+X_{R}\left(t\right)+\left(y-x\right)\times rand$$

(7) $$L\overset{´}{e}vy\left(D\right)=s\times \frac{u\times \sigma }{{\left|v\right|}^{\frac{1}{\beta }}}$$

(8) $$\sigma =(\frac{\Gamma \left(1+\beta \right)\times sine\left(\frac{\pi \beta }{2}\right)}{\Gamma \left(\frac{1+\beta }{2}\right)\times \beta \times 2^{\frac{\beta -1}{2}}})$$

(9) x=r×sin(θ)

(10) y=r×cos(θ)

(11) $$r=r_{1}+U\times D_{1}$$

(12) $$\theta =-\omega \times D_{1}+\frac{3\times \pi }{2}$$

where $X_{i}\left(t+1\right)$ is the position of the *i*-th individual at the (t+1)-th iteration. $L\overset{´}{e}vy\left(D\right)$ is the L$\overset{´}{e}$vy flight distribution function and *D* represents the dimensional space, specifically calculated by Equation (7). $X_{R}\left(t\right)$ is the random individual sampled in the range [1,N] at the *i*-th iteration. *x* and *y* are computed by Equations (9) and (10), respectively.

In Equation (7), *s* is a constant with a value of 0.01. *u* and *v* are random numbers ranging from 0 to 1. σ is calculated by Equation (8). In Equations (9) and (10), *r* is calculated by Equation (11), θ is calculated by Equation (12), $r_{1}$ is used to fix the count of search cycles, whose value is taken between 1 and 20, and *U* is a small amount that is fixed at 0.00565. ω is a small value of 0.005, and $D_{1}$ is an integer from 1 to the length of the search space (Dim).

The third strategy is mainly based on Aquila’s low flying and slow falling attacks. This action is expressed mathematically as Equation (13):

(13) $$X_{i}\left(t+1\right)=\left(X_{best}\left(t\right)-X_{M}\left(t\right)\right)\times \alpha -rand+\left(\left(UB-LB\right)\times rand+LB\right)\times \delta$$

where δ and α denote the adjustment parameters of the exploitation process.

The fourth strategy refers to Aquila’s behavior of walking and catching prey. This behavior is mathematically shown in Equation (14):

(14) $$X_{i}\left(t+1\right)=QF\times X_{best}\left(t\right)-\left(G_{1}\times X\left(t\right)\times rand\right)-G_{2}\times L\overset{´}{e}vy\left(D\right)+rand\times G_{1}$$

(15) $$QF\left(t\right)=t^{\frac{2\times rand-1}{{\left(1-T\right)}^{2}}}$$

(16) $$G_{1}=2\times rand-1$$

(17) $$G_{2}=2\times \left(1-\frac{t}{T}\right)$$

where Equation (15) is the QF expression. Equations (16) and (17) represent $G_{1}$ and $G_{2}$, respectively. $L\overset{´}{e}vy\left(D\right)$ is the L$\overset{´}{e}$vy flight distribution function calculated using Equation (7). *t* and *T* present the current iteration and the maximum number of iterations respectively.

## 3. The Proposed Algorithm

This section introduces the main parts of our proposed algorithm separately, mainly including six parts: convolutional layer representation, the block structure, the grouping strategy, the position update representation, the fitness representation, and the decoding.

### 3.1. Convolutional Layer Representation

When we consider the design of algorithms that handle complex structures (e.g., CNN architectures), it is important to choose an appropriate encoding method to represent the CNN architecture and to avoid additional hard-coded management rules to ensure a reasonable construction of the architecture. In this paper, we directly represent each block in the CNN with a binary encoding and use evolutionary computation to update the encoding for the purpose of tuning the neural network architecture. For each training, testing, and evaluation using CNN, the model is compiled from an array containing the details of each layer in the particle. However, only different CNN structures can be found using this method. Therefore, we search the relevant hyperparameters of each layer at the same time.

We use a binary expression to represent the actual meaning of what each layer represents. Each of the different types of the layer is represented by a three-bit binary encoding, with three bits representing the eight possible options, each containing at least one activation-function layer, two convolutional layers, and one regularization layer.

Where the binary number ‘000’ indicates that the average pooling layer is selected; ‘001’ indicates we use the maximum pooling layer in the architecture; ‘010’ indicates that the skip-connection layer is used; ‘011’ indicates the selection of a convolutional layer with a convolutional kernel size of 3×3 and a padding value of one; ‘100’ means a convolutional layer with a kernel size of 5×5 and a padding value of two; ‘101’ shows a convolutional layer whose kernel size is 7×7 and padding value is four; ‘110’ implies choosing a convolutional layer having a kernel size of 3×3, a padding value of two, and a dilation size of two; and ‘111’ indicates selecting a convolutional layer with a kernel size of 5×5, a padding value of four, and a dilation size of two. The specific representation is shown in Table 1. The details of the operation will be shown in Algorithm 1.
**Algorithm 1** The pseudo code of HAGCNN**Initialize:** The dimension of each individual is *D*, and each individual $P_{i}$ is initialized with *D* random numbers, i∈[1,N]. $f(P_{i})$ represents the fitness function of the individual $P_{i}$, The current number of iterations is T=1, the maximum number of iterations is *M*, and *R* is the number of iterations executing the communication strategy.**Iteration:**1:**while**T<M**do**2:    **for** i=1 to *N* **do**3:        Decode the individual and generate the corresponding network.4:        Train the network using the training dataset.5:        The network is tested on the test data set, and the accuracy is returned as the fitness value.6:    **for** i=1 to *N* **do**7:        Update the mean value of the current solution $X_{M}\left(t\right)$.8:        **if** $t\leq (\frac{2}{3})\times T$ **then**9:           **if** rand≤0.5 **then**10:               $P_{i}$ is performed **OR** operate bit by bit with $P_{best}$.11:               The results were mutated randomly.12:               Then cross operate the mutation result with $P_{best}$.13:               The encoding of the particle representation is divided into *n* groups and a pooling layer is inserted in the last position of each group that satisfies the condition.14:               Calculate the fitness value of the new individual. If the value is better than $P_{best}$, update $P_{best}$ with the individual.15:           **else**16:               Random mutation of $P_{best}$ into several bits.17:               The encoding of the particle representation is divided into *n* groups and a pooling layer is inserted in the last position of each group that satisfies the condition.18:               Calculate the fitness value of the new individual. If the value is better than $P_{best}$, update $P_{best}$ with the individual.19:        **else**20:           **if** rand≤0.5 **then**21:               Update the mean value of the current solution $P_{M}$.22:               The optimal individual $P_{best}$ and the average individual $P_{M}$ are **XOR**ed.23:               The result of **XOR** is crossed with random binary coding, and the crossing position is controlled to be in the second half of the coding.24:               The encoding of the particle representation is divided into *n* groups and a pooling layer is inserted in the last position of each group that satisfies the condition.25:               Calculate the fitness value of the new individual. If the value is better than $P_{best}$, update $P_{best}$ with the individual.26:           **else**27:               Random mutation of $P_{best}$ into several bits, and the number of mutations should not exceed half of the total number.**Output:** The global best individual $P_{best}$, and the best fitness value $f(P_{best})$.

Since the particle length is fixed after initialization, to search the CNN structure at variable length and to increase the possibility of searching, this paper achieves this purpose by using the inclusion of a skip-connection layer by setting the skip-connection layer to be denoted by ‘010’; if this layer is selected, the output of the previous layer is copied directly to the next layer, which is equivalent to reducing the actual number of valid layers in the whole network by one layer and thus achieving a variable length search.

The number of output channels per convolutional layer is another parameter that has a large impact on the network performance. Therefore, our algorithm also includes this parameter in the search space. Similarly, we convert the number of output channels of the convolutional layers to binary and concatenate it to the convolution type. Thus, our encoding string becomes a L×(3+X)-bit binary string, where a complete convolution block is represented by 3+X bits, which contain three bits of binary for the convolution type and *X* bits for the number of output channels of the layer. Here *L* denotes the number of layers present in the network and *X* remains fixed during a search.

### 3.2. Block Structure

**Figure 2:**
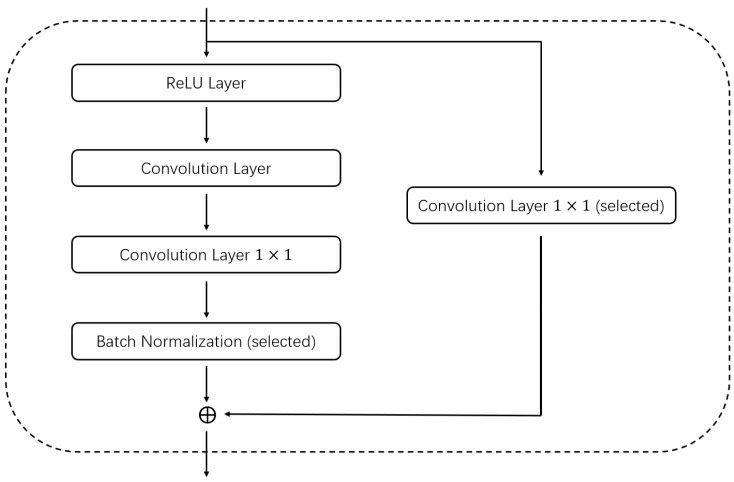
Basic block structure.

**Figure 3:**
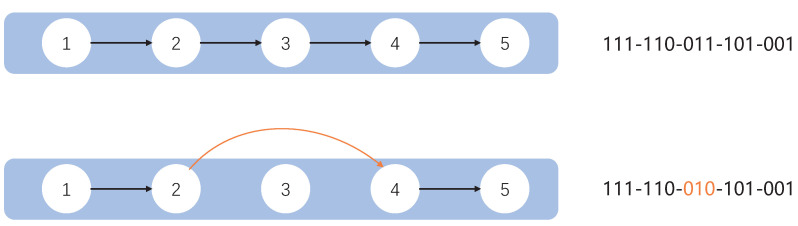
Example of the network’s encoding by different codings.

In past studies, researchers have often combined a number of fixed layers together to form block structures, and using such block structures can dramatically improve the performance of the network. In this article, we also used a similar structure. We combine the convolution layer, batch-normalization layer, and activation layer into a base convolution block. Furthermore, when encoding, we encode only the convolution block and not the separate convolution layers. When combining these layers, we consider both the convolution kernel size, stride size, padding, and dilation to ensure that the resolution of the input block is the same as the resolution of the block output. To further improve the performance of the convolution block, we incorporate the idea of a residual block, and our convolution block similarly copies the input and adds it to the output of the convolution layer. We add a 1×1 convolution layer for channel fusion so that the number of input channels is the same as the number of output channels for direct summation for blocks where the number of input channels is different from the number of output channels. For example, Figure 2 shows a basic block structure. An example of a network with different encoding methods after combining different block structures is shown in Figure 3. As shown in Figure 3, the numbers indicate the basic block structures, and the corresponding codes are shown after each structure.

### 3.3. Grouping Strategy

**Figure 4:**
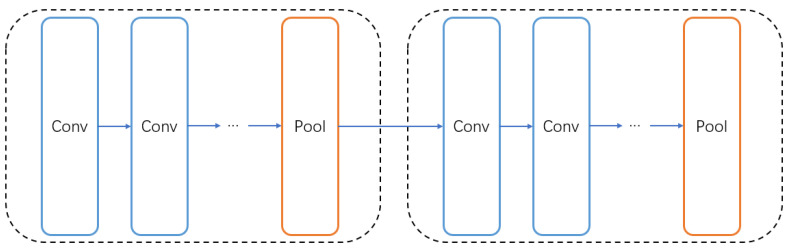
The example of grouping strategy.

In order to generate better architectures, we group the searched architectures. The purpose of grouping is to reasonably intersperse the pooling layers in the network structure to make our network achieve better results. We divide all the layers into *n* groups, assuming that the whole network contains *L* layers (including skip connections), there are L/n layers in each group, and we detect whether the last layer in each group is a pooling layer to avoid adding redundant pooling layers repeatedly. Figure 4 shows an example of grouping the entire network structure, and we make sure that the last layer of each group is the pooling layer.

### 3.4. Location Update Representation

After representing the CNN structure with a string of binary characters, this string is used as input to the evolutionary computation algorithm formula, and several operations are performed during the bring-up process. Algorithm 1 further describes this part of the specific process.

#### 3.4.1. Crossover Operation

**Figure 5:**
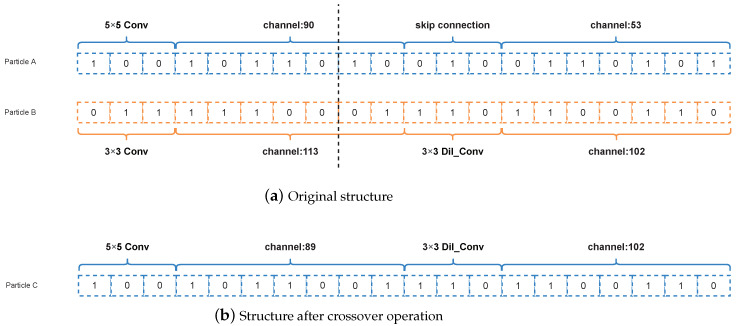
Example of the crossover operation between two particles.

If the current particle position is multiplied by an arbitrary constant which is more than 1 in the formula, the algorithm performs a crossover operation. Note that it is rounded upwards to obtain the final constant if the current particle position is multiplied by a fractional number. Furthermore, we assume the length of the constant part is *C* and the length of the non-constant part is N−C. Then, the two coded strings are first truncated according to the constant digit and we cross-patch the two intercepted strings following the behavior of the chromosome crossover. Assuming the code *A* is $code_{A}=\left[c_{a,1},c_{a,2},\cdots ,c_{a,i},c_{a,i+1},\cdots ,c_{a,N}\right]$, and the code *B* is $code_{B}=\left[c_{b,1},c_{b,2},\cdots ,c_{b,i},c_{b,i+1},\cdots ,c_{b,N}\right]$. If the truncation is from the *i*-th bit, the results after crossing $code_{A}$ and $code_{B}$ are as follows, $code_{A}=\left[c_{a,1},c_{a,2},\cdots ,c_{a,i}{,}_{b,i+1},\cdots ,c_{b,N}\right]$, $code_{B}=\left[c_{b,1},c_{b,2},\cdots ,c_{b,i},c_{a,i+1},\cdots ,c_{a,N}\right]$. The specific operation is shown in Figure 5. Figure 5a represents the initial structure of any two individuals and Figure 5b represents the encoded structure of two individuals after the crossover operation.

#### 3.4.2. Mutation Operations

##### Mutation Operation of Random Number

**Figure 6:**
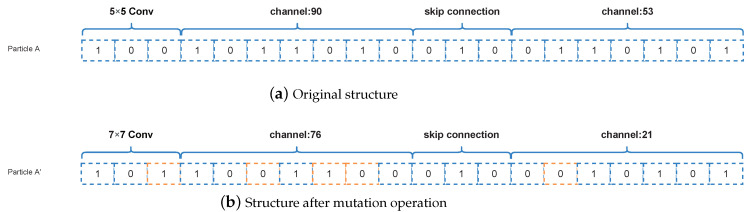
Example about the mutation operation of random number.

If the current particle position is multiplied by a random number from zero to one, the algorithm performs a mutation operation. Here we use a random number that is subject to a uniform distribution. Furthermore, the bits 0 to dim are randomly selected for the mutation. In this process, the parameter dim represents the dimension of the algorithm. The specific mutation is that the original 0 bit becomes 1 and the 1 bit becomes 0 for the mutation operation. Assuming a code of $code_{A}=\left[1,0,1\right]$, we randomly selected 2 bits for mutation. The result may be [0,1,1], [1,1,0], or [0,1,0]. The specific representation is shown in Figure 6. Figure 6a represents the original structure before the mutation operations and Figure 6b represents the structure after the mutation operations, where the orange part in Figure 6b is the coding of the mutation.

##### Mutation Operation of L$\overset{´}{e}$vy Flight

If the current particle position is multiplied by a L$\overset{´}{e}$vy flight [34,35] operand in the formula, the CNN structure corresponding to the best CNN structure is encoded as a mutation operation. The specific variation is performed in the same way as the random number mutation operation.

#### 3.4.3. Selection Operation

If two particle positions are added or subtracted in the formula, the selection operation is performed. In this case, if the two-particle positions are subtracted, the operation is the Bitwise Exclusive OR operation. If the two-particle positions are added, the operation is the Bitwise OR operation.

##### Bitwise Exclusive OR

**Figure 7:**
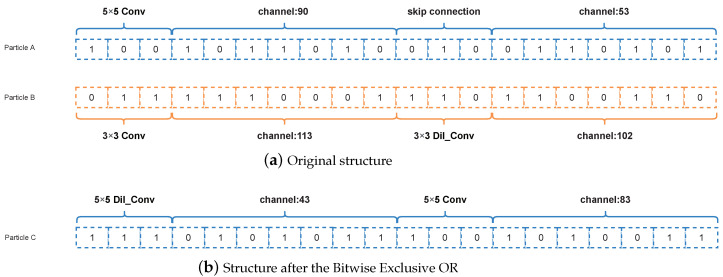
Example about selection operation of the Bitwise Exclusive OR.

The operation of the Bitwise Exclusive OR is an operation in which two numbers participating in an operation are converted to binary (0,1) and then subjected to the Exclusive OR operation, as long as 1 is given when the numbers in the corresponding bits are not the same and 0 when they are the same. Assuming the two codes are $code_{A}=\left[1,0,0,1,0\right]$, $code_{B}=\left[0,0,1,0,0\right]$, the result is [1,0,1,1,0]. The details of the relevant operation are more prominently represented in Figure 7, where particle *A* and particle *B* represent the positions of two random particles. When the positions of the two particles are subtracted, the Bitwise Exclusive OR operation is performed, the result is shown in Figure 7b.

##### Bitwise OR

**Figure 8:**
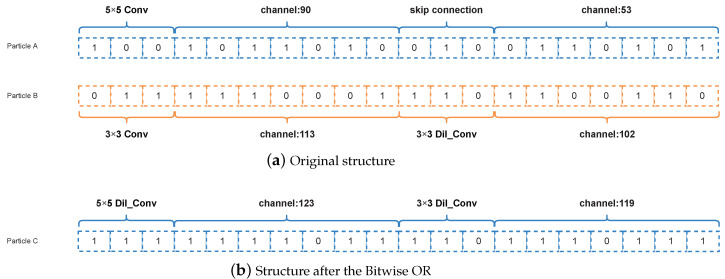
Example about selection operation of the Bitwise OR.

The Bitwise OR is similar to the Bitwise Exclusive OR, in which is the AND operation is changed to OR operation. When the two bits are 0, the bit is 0, the rest of the cases the bit is 1. Assuming the two codes are $code_{A}=\left[1,0,0,1,0\right]$, $code_{B}=\left[0,0,1,0,0\right]$, the result is [1,0,1,1,0]. Similarly, when the positions of the two particles are added together, the Bitwise OR operation is performed, the result is shown in Figure 8b.

### 3.5. Fitness Evaluation

After iterations of the algorithm, the encoding will change and the changed encoding still corresponds to specific network architecture. The network uses the Adam optimizer to adjust the trainable parameters in the network and *CosineAnnealing* is used to adjust the learning rate. For the first 10 epochs we let the learning rate decrease from a predetermined maximum to a predetermined minimum, and for the last 10 epochs we let the learning rate increase from the minimum to the maximum. To speed up the network training, each network structure searched by the algorithm during the iterative process is set to an epoch value of one. When the iteration of the algorithm is completed, the epoch value used for training the best-obtained network structure is set to 100. The loss function used for training is set to the cross-entropy loss, and the return value of this function is used as the fitness value. The return value of this function is used as the fitness value to guide the execution of the evolutionary computation algorithm. Finally, we transfer part of the training set as input to the network, train the network, and return the fitness value.

### 3.6. Decoding

According to Table 1, the different binary strings can be decoded into the corresponding layer and the corresponding parameter information, and these strings represent the parameter values for this layer. After decoding all the interfaces, the final CNN structure is achieved by concatenating all the decoding layers in the best global case. By concatenating all decoding layers in the same order as the interfaces in the particle vector, the final CNN architecture can be obtained.

## 4. Experimental Preparation

Our experiments are based on the NVIDIA RTX 3090, with an Intel Xeon Gold 6226R CPU @ 2.90 GHz.

### 4.1. Datasets

Considering the cost of time consumption during NAS processing, and the limitation of our computing device resources, we only select datasets with small input sizes for testing. We select two basic datasets to evaluate the final results, namely MNIST and CIFAR-10 datasets.

Specifically, the MNIST [36,37] dataset contains images of handwritten numbers from 0 to 9. Each sample is a 28×28 pixel. A total of 60,000 training samples and 10,000 test samples are contained in the dataset.

CIFAR-10 [38,39] is a computer vision dataset for universal object recognition; it is gathered by Hinton’s students Alex Krizhevsky and Ilya Sutskever. It contains 60,000 32×32 RGB color images with a total of 10 classifications. All photos are divided into 10 different categories, namely ‘airplane’, ‘bird’, ‘cat’, ‘automobile’, ‘dog’, ‘frog’, ‘horse’, ‘deer’, ‘ship’, and ‘truck’. Of these, 50,000 are used for the training set and 10,000 for the test set. Specific datasets information is shown in Table 2.

### 4.2. Parameter Setting

The parameter setting in this paper is divided into two main parts—on the algorithm side and the training side—the details of which are given in Table 3.

Among them, the population size and the number of iterations are used for the setting of the evolutionary algorithm. The population size indicates the number of networks tested in one iteration and the number of iterations limits the maximum iterations of the algorithm. The number of iterations limits the maximum number of iterations of the algorithm.

In addition, the main parameters are batch size, dropout rate, epochs for particle evaluation, and epochs for the global best in the CNN training process. Batch size controls the amount of data fed into the model for one training. The dropout rate controls the rate at which fully connected layers randomly discard neurons in a single training session to prevent overfitting of the network. The epochs for particle evaluation are the number of times the training set is reused in a single training session. Using lower epochs for particle evaluation can significantly reduce the training time with little impact on the overall algorithm performance. The epochs for the global best is the number at which the training set is repeatedly used when training the network represented by that individual after the optimal individual has been found. Therefore, moderate values need to be selected to train the network. Note that the trained network will not be capable of achieving the desired result if the value is too small. Similarly, a large value may lead to overfitting. Specific information is shown in detail in Table 3.

### 4.3. Baseline Model

#### 4.3.1. On the MNIST Dataset

For better evaluation of the performance of our model, we select the manually designed networks LeNet-5 [6], ResNet [8,40], CapsNet [41], and DropConnect [42], and the automatically designed network models EvoCNN [14], MetaQNN [12], IPPSO [15], psoCNN [19], and GeNet [43] as baseline models on the MNIST dataset, and compare them with our proposed HAGCNN model in terms of test-set error rate, and the number of parameters and running speed-related metrics are compared with our proposed model.

Firstly, we compare the first convolutional neural network LeNet-5. Although they are now obsolete, a comparison is still necessary. Secondly, the EvoCNN automatic generation model, which is based on the GA, is chosen for comparison. In addition, MetaQNN-based reinforcement learning is used for performance comparison. Additionally, ResNet, CapsNet, and DropConnect are used as a benchmark model for comparison.

Furthermore, IPPSO is the first model to use the PSO algorithm for automatic CNN generation. It is mainly inspired by IP addresses, by mapping attribute data to the corresponding IP address ranges and processing them to obtain the optimal CNN model. Besides, the psoCNN model is used for comparison, combining CNN generation with the PSO algorithm by designing a novel velocity-position calculation. Additionally, it used 10 iterations on 20 particles for experiments, and the experimental results proved to have better experimental results.

#### 4.3.2. On the CIFAR-10 Dataset

On the CIFAR-10 dataset, we select the manually designed networks VGGNet [44], ResNet [8], and DensNet [45] and the automatically designed network models MetaQNN [12], NASNet [13], GeNet [43], CoDeepNEAT [46], and Large-Scale Evolution [47] as benchmark models to compare with our proposed HAGCNN model in terms of test-set error rate, parameter footprint, and running speed-related metrics.

VGGNet is a deep convolutional neural network developed by the Oxford University Computer Vision Portfolio together with researchers at Google DeepMind. In 2014, it was runner-up in the ILSVRC competition and winner of the localization project, with an error rate of 7.5 percent on top 5. Meanwhile, DenseNet starts with features and achieves better results and fewer parameters by making the best use of the feature.

NASNet, which no longer requires relevant experts to build convolutional network architectures with human knowledge, directly uses RNN to compute the hyperparameter and realize AI automatic learning, CoDeepNEAT, which optimizes the deep learning architecture through evolution. Through the extension of existing neuroevolutionary methods in terms of components, topology, and hyperparameters, the approach achieves results comparable to the best human designs in standard baselines in language modeling and object recognition.

Large-Scale Evolution modified the evolutionary algorithm so that it can be used for NAS, and it is worth noting that the neural network architecture it searches has no fixed network depth.

## 5. Experiment and Analysis

To have a clearer view of the effect of the proposed model, we analyze the effect of the proposed algorithm from five perspectives: experimental results, comparison experiment with baseline model, visualization of experimental results, comparison of the effect of random evolution and the proposed algorithm, and the change of the algorithm position with the number of iterations, respectively.

### 5.1. Experimental Results

We search through 20 particles with 10 iterations in the search space and train the network structure obtained from the search with 100 epochs and 200 epochs on the MNIST and CIFAR-10 datasets, respectively. We compare the performance of the best CNN structure searched by our proposed algorithm in terms of error rate, parameters, and search speed.

The experimental results show that the automatically generated CNN model reached an error rate of 0.31% on the MNIST dataset with parameters 1.33M and a search speed of 0.5 h/1 GPU.

Experimental results based on the CIFAR-10 dataset show that the automatically generated CNN model has an error rate of 6.82% with 10.7M parameters and a search speed of 12 h/1 GPU. To find a better architecture, the parameters used are different from those used in the experiment on the MNIST dataset. When we search the optimal network architecture for the MNIST dataset, we conduct 1-epoch training on the searched network. In CIFAR-10 dataset search architecture, the network accuracy after 1-epoch training can not guide the evolutionary computing algorithm to find a better structure, which makes the evolutionary computing algorithm fall into local optimization. Therefore, we train 20 epochs for each searched network and return the obtained accuracy as a fitness value to the evolutionary computing algorithm. Similarly, because it is more difficult to train the network on the CIFAR-10 dataset than on the MNIST dataset, it needs more rounds of training to obtain a satisfactory accuracy. Therefore, the optimal architecture that can be found at the end of the iteration of the evolutionary computing algorithm is trained 200 epochs.

### 5.2. Comparison Experiment with Baseline Model

On the MINIST dataset, we compare our obtained CNN model with the current CNN network-generated models in manual and automatic modes, respectively. As shown in Table 4, firstly, our model results in an error rate on the test set that exceeds the baseline LeNet-5 and ResNet, and it is also near to the results of the two advanced CapsNet and DropConnect. In the comparison with the automatically generated model, we can find that our proposed model is not only lower in error than IPPSO, psoCNN, and GeNet, but also lower in the number of parameters than the currently known IPPSO and psoCNN in terms of the number of parameters, and nearly three times the number of psoCNN parameters. Additionally, the speed of searching for the optimal structure is much higher than that of the three known NAS models. This indicates that our proposed algorithm can find a better architecture for the image classification task.

On the CIFAR-10 dataset, we compare the results of the proposed algorithm with those of current state-of-the-art neural architecture search algorithms. As shown in Table 5, although our proposed algorithm still has a large gap from these advanced search models in the item of error rate, we only beat VGGNet, GeNet, and CoDeepNEAT, but compared to MetaQNN, NASNet, and Large-Scale Evolution, we spend substantially less time to. Therefore, our proposed algorithm still has practical significance.

### 5.3. Visualisation of Results

To explore the trajectory trends of the particles in the evolutionary computation, we visualize the particles that varied with the number of iterations. We chose the principal component analysis (PCA) [48,49] method, which selects the two most important components from all the particle dimensions to form a three-dimensional landscape.

**Figure 9:**
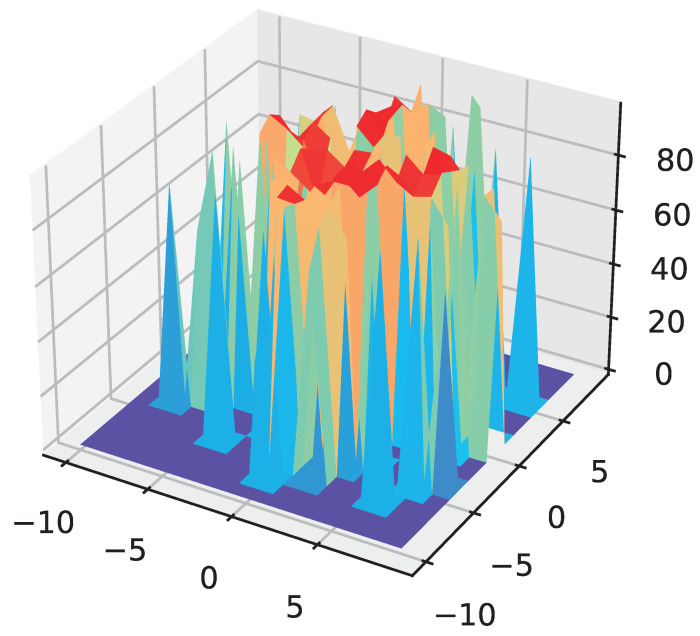
The particle position visualization.

As shown in Figure 9, the *x* and *y* axes indicate the two extracted dimensions respectively, while the *z* axis indicates the accuracy of the network corresponding to that particle on the test set. This visualization shows the accuracy of the network obtained after the 1 epoch of training for different particle positions.

**Figure 10:**
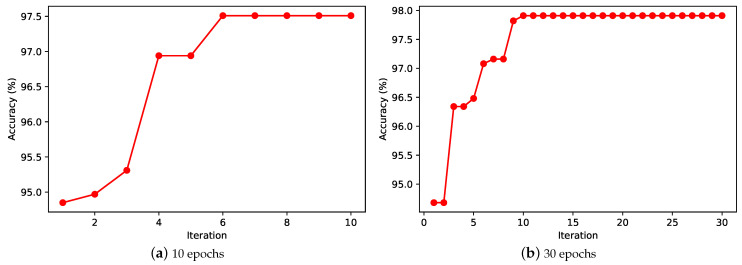
Results on the MNIST test set.

To investigate the effect of the number of iterations on the search algorithm, we delay the number of iterations to 30 and re-run the experiment. The results are shown in Figure 10. Under the result of 1-epoch training, the horizontal coordinate indicates the current number of iterations and the vertical coordinate indicates the best network accuracy on the test set that can be found in that iteration. When the number of iterations is 10, our proposed algorithm converges in the 6-th generation, and when the number of iterations is extended to 30, the algorithm still converges within 10 generations. This indicates that our proposed algorithm can converge to the optimal value quickly. This also means that our proposed algorithm is applicable to solve the neural architecture search problem.

### 5.4. Comparison Experiment between Random Evolution and Proposed Algorithm

**Figure 11:**
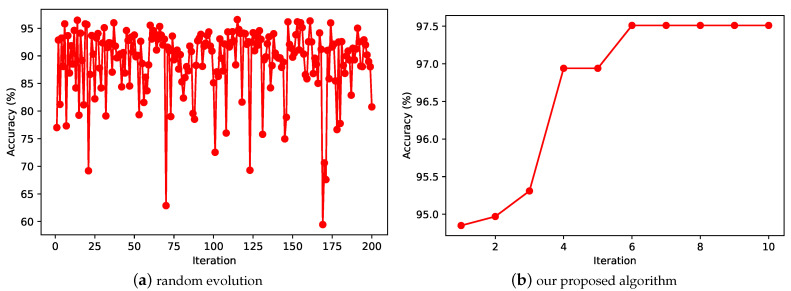
Comparison of the results of random evolution and our proposed algorithm computation on the MNIST dataset.

As shown in Figure 11, Figure 11a shows the accuracy of the test set using the random evolution approach on the MNIST dataset using 20 particles with 10 iterations, and Figure 11b shows the accuracy of the test set obtained using the proposed model on the MNIST dataset under the same conditions. The comparison of the two subplots in the figure shows that our proposed CNN evolution model has a significant improvement, finally reaching 99.7% on the MNIST dataset, and using random evolution can only reach 96.5% on the MNIST dataset. We can also see from the figure that random search exhibits a high degree of disorder. The evolutionary computation algorithm, on the other hand, can use the information from the previous iteration to search for better network architecture in a more optimal direction. This shows the great advantage of evolutionary computing in searching neural network architectures. We believe that if we set up the algorithm using a larger number of iterations, we may have better evolutionary results.

### 5.5. Position Movement Analysis of Particles from One Generation to Another

**Figure 12:**
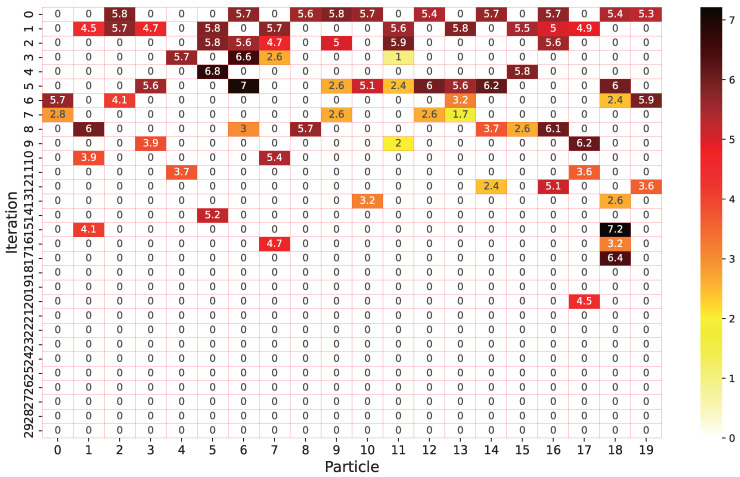
The distance of each generation of particles from its position in the previous generation.

To observe the patterns of the network architecture found by our proposed algorithm, we study the evolution process, how each particle changed in each generation, and compare the distance each particle moved in each generation. The result is shown in Figure 12. In this part of the experiment, we record the position of each particle after each iteration and calculated the distance of all particles from their positions after the previous iteration. According to the experimental data, in all 30 iterations, the average distance moved by each particle is 17.8, the maximum distance moved is 7.21, and the minimum distance moved is 1. On average, each particle moved 3.45 times during all iterations. This shows that the proposed algorithm can effectively explore the entire architecture space.

## 6. Conclusions and Future Works

In this paper, we present a novel deep architecture generation model based on the AO and GA. The framework performs optimal architectural searches in a specific search space. By combining hybrid binary coding and particle-layer locations, and adding skip connections and residual blocks to increase the range of searchable space, a high-accuracy and low-time-cost automatic generation model is finally obtained, and the computing cost of NAS has been reduced to an acceptable level. In future work, the inclusion of more block structures similar to residual blocks and the combination of search directions with a topology search can be considered to increase the possibility of searching for solutions. Additionally, the direct search can be performed with multi-objectives under the simultaneous consideration of objectives, such as time cost and delay rate, to enrich our search model. Meanwhile, we can consider applying the model to more areas for further research.

## Figures and Tables

**Table 1:** Convolutional layer representation.

Coding	Type of Layer	Parameter
‘000’	average pooling layer	kernel size = 3×3
‘001’	maximum pooling layer	kernel size = 3×3
‘010’	skip connection	
‘011’	convolutional layer	kernel size = 3×3
		padding = 1
‘100’	convolutional layer	kernel size = 5×5
		padding = 2
‘101’	convolutional layer	kernel size = 7×7
		padding = 4
‘110’	convolutional layer	kernel size = 3×3
		padding = 2
		dilation size = 2
‘111’	convolutional layer	kernel size = 5×5
		padding = 4
		dilation size = 2

**Table 2:** Summary of the datasets.

Dataset	Input Size	Training	Test	Classes
MNIST	28×28×1	60,000	10,000	10
CIFAR-10	32×32×1	50,000	10,000	10

**Table 3:** Summary of the parameters.

Parameter	Value
**HAGCNN Optimizer**	
population size	20
number of iterations	10
**CNN training(MNIST)**	
bath size	240
dropout	0.5
epochs for particle evaluation	1
epochs for the global best	100
**CNN training(CIFAR-10)**	
bath size	128
dropout	0.5
epochs for particle evaluation	20
epochs for the global best	200

**Table 4:** Comparison with baseline model on the MNIST dataset.

Baseline Model	Test Error (%)	Params (MB)	Cost
LeNet-5	0.75	0.43	-
ResNet	0.41	-	-
CapsNet	0.25	-	-
DropConnect	0.21	-	-
IPPSO	1.12	1.47	2 GPUs/2.5 h
psoCNN	0.44	3.26	1 GPU/7.3 h
GeNet	0.38	-	10 GPUs/2 days
HAGCNN	0.31	1.33	1 GPU/0.5 h

**Table 5:** Comparison with baseline model on the CIFAR-10 dataset.

Baseline Model	Test Error (%)	Params (MB)	Cost
VGGNet	7.25	-	-
ResNet (depth = 110)	6.43	1.7	-
DensNet (depth = 100)	4.10	7.0	-
MetaQNN	6.92	11.2	10 GPUs/8 days
NASNet	4.47	7.1	500 GPUs/33 days
GeNet	7.10	-	10 GPUs/17 days
CoDeepNEAT	7.30	-	-
Large-Scale Evolution	5.40	5.4	-/250 h
HAGCNN	6.82	10.7	1 GPU/12 h

## Data Availability

Not applicable.

## References

[1] Lu D., Weng Q. (2007). A survey of image classification methods and techniques for improving classification performance. Int. J. Remote Sens..

[2] Huang K.W., Lin C.C., Lee Y.M., Wu Z.X. (2019). A deep learning and image recognition system for image recognition. Data Sci. Pattern Recognit..

[3] Liao T.L., Chen H.C., Yan J.J. (2020). Design of Real-time Face Position Tracking and Gesture Recognition System based on Image Segmentation Algorithm. J. Netw. Intell..

[4] Nadkarni P.M., Ohno-Machado L., Chapman W.W. (2011). Natural language processing: An introduction. J. Am. Med. Inform. Assoc..

[5] Spyns P. (1996). Natural language processing in medicine: An overview. Methods Inf. Med..

[6] LeCun Y., Bottou L., Bengio Y., Haffner P. (1998). Gradient-based learning applied to document recognition. Proc. IEEE.

[7] Krizhevsky A., Sutskever I., Hinton G.E. (2012). Imagenet classification with deep convolutional neural networks. Adv. Neural Inf. Process. Syst..

[8] He K., Zhang X., Ren S., Sun J. Deep residual learning for image recognition. Proceedings of the IEEE Conference on Computer Vision and Pattern Recognition (CVPR).

[9] Stanley K.O., D’Ambrosio D.B., Gauci J. (2009). A hypercube-based encoding for evolving large-scale neural networks. Artif. Life.

[10] Zoph B., Le Q.V. (2016). Neural architecture search with reinforcement learning. arXiv.

[11] Fan Z., Hu G., Sun X., Wang G., Dong J., Su C. (2022). Self-attention neural architecture search for semantic image segmentation. Knowl.-Based Syst..

[12] Baker B., Gupta O., Naik N., Raskar R. (2016). Designing neural network architectures using reinforcement learning. arXiv.

[13] Zoph B., Vasudevan V., Shlens J., Le Q.V. Learning Transferable Architectures for Scalable Image Recognition. Proceedings of the IEEE Conference on Computer Vision and Pattern Recognition (CVPR).

[14] Sun Y., Xue B., Zhang M., Yen G.G. (2019). Evolving deep convolutional neural networks for image classification. IEEE Trans. Evol. Comput..

[15] Wang B., Sun Y., Xue B., Zhang M. Evolving deep convolutional neural networks by variable-length particle swarm optimization for image classification. Proceedings of the 2018 IEEE Congress on Evolutionary Computation (CEC).

[16] Kennedy J., Eberhart R. Particle swarm optimization. Proceedings of the ICNN’95-International Conference on Neural Networks.

[17] Chu S.C., Du Z.G., Peng Y.J., Pan J.S. (2021). Fuzzy Hierarchical Surrogate Assists Probabilistic Particle Swarm Optimization for expensive high dimensional problem. Knowl.-Based Syst..

[18] Wang H., Sun H., Li C., Rahnamayan S., Pan J.s. (2013). Diversity enhanced particle swarm optimization with neighborhood search. Inf. Sci..

[19] Junior F.E.F., Yen G.G. (2019). Particle swarm optimization of deep neural networks architectures for image classification. Swarm Evol. Comput..

[20] Lawrence T., Zhang L., Lim C.P., Phillips E.J. (2021). Particle swarm optimization for automatically evolving convolutional neural networks for image classification. IEEE Access.

[21] Pham H., Guan M., Zoph B., Le Q., Dean J. Efficient neural architecture search via parameters sharing. Proceedings of the International Conference on Machine Learning.

[22] Li C., Peng J., Yuan L., Wang G., Liang X., Lin L., Chang X. Block-wisely supervised neural architecture search with knowledge distillation. Proceedings of the IEEE/CVF Conference on Computer Vision and Pattern Recognition.

[23] Fukushima K., Miyake S. (1982). Neocognitron: A Self-Organizing Neural Network Model for a Mechanism of Visual Pattern Recognition. Competition and Cooperation in Neural Nets.

[24] Yamashita R., Nishio M., Do R.K.G., Togashi K. (2018). Convolutional Neural Networks: An Overview and Application in Radiology. Insights Into Imaging.

[25] Liu X., Li J., Hu C., Pan J.S. Deep convolutional neural networks-based age and gender classification with facial images. Proceedings of the 2017 First International Conference on Electronics Instrumentation & Information Systems (EIIS).

[26] Shi Y., Zhu Y.Y., Fang J., Li Z.S. (2021). Pose Measurement of Excavator Based on Convolutional Neural Network. J. Netw. Intell..

[27] Botalb A., Moinuddin M., Al-Saggaf U., Ali S.S. Contrasting Convolutional Neural Network (CNN) with Multi-Layer Perceptron (MLP) for Big Data Analysis. Proceedings of the 2018 International Conference on Intelligent and Advanced System (ICIAS).

[28] Singh G., Sachan M. Multi-layer perceptron (MLP) neural network technique for offline handwritten Gurmukhi character recognition. Proceedings of the 2014 IEEE International Conference on Computational Intelligence and Computing Research.

[29] Arora S., Bhattacharjee D., Nasipuri M., Basu D.K., Kundu M. (2011). Complementary features combined in a MLP-based system to recognize handwritten devnagari character. J. Inf. Hiding Multimed. Signal Process..

[30] Mirjalili S. (2019). Genetic algorithm. Evolutionary Algorithms and Neural Networks.

[31] Pan J.S., Kong L., Sung T.W., Tsai P.W., Snášel V. (2018). A Clustering Scheme for Wireless Sensor Networks based on Genetic Algorithm and Dominating Set. J. Internet Technol..

[32] Abualigah L., Yousri D., Abd Elaziz M., Ewees A.A., Al-Qaness M.A., Gandomi A.H. (2021). Aquila optimizer: A novel meta-heuristic optimization algorithm. Comput. Ind. Eng..

[33] Wang S., Jia H., Abualigah L., Liu Q., Zheng R. (2021). An Improved Hybrid Aquila Optimizer and Harris Hawks Algorithm for Solving Industrial Engineering Optimization Problems. Processes.

[34] Haklı H., Uğuz H. (2014). A novel particle swarm optimization algorithm with Levy flight. Appl. Soft Comput..

[35] Song P.C., Pan J.S., Chu S.C. (2020). A parallel compact cuckoo search algorithm for three-dimensional path planning. Appl. Soft Comput..

[36] LeCun Y., Boser B., Denker J.S., Henderson D., Howard R.E., Hubbard W., Jackel L.D. (1989). Backpropagation applied to handwritten zip code recognition. Neural Comput..

[37] Deng L. (2012). The MNIST Database of Handwritten Digit Images for Machine Learning Research [Best of the Web]. IEEE Signal Process. Mag..

[38] Abouelnaga Y., Ali O.S., Rady H., Moustafa M. CIFAR-10: KNN-Based Ensemble of Classifiers. Proceedings of the 2016 International Conference on Computational Science and Computational Intelligence (CSCI).

[39] Li H., Liu H., Ji X., Li G., Shi L. (2017). CIFAR10-DVS: An Event-Stream Dataset for Object Classification. Front. Neurosci..

[40] Allen-Zhu Z., Li Y. What Can ResNet Learn Efficiently, Going Beyond Kernels?. Proceedings of the 33rd International Conference on Neural Information Processing Systems.

[41] Sabour S., Frosst N., Hinton G.E. Dynamic routing between capsules. Proceedings of the 31st International Conference on Neural Information Processing Systems.

[42] Wan L., Zeiler M., Zhang S., Le Cun Y., Fergus R. Regularization of neural networks using dropconnect. Proceedings of the 30th International Conference on Machine Learning.

[43] Xie L., Yuille A. Genetic CNN. Proceedings of the IEEE International Conference on Computer Vision (ICCV).

[44] Simonyan K., Zisserman A. (2014). Very deep convolutional networks for large-scale image recognition. arXiv.

[45] Huang G., Liu Z., Van Der Maaten L., Weinberger K.Q. Densely Connected Convolutional Networks. Proceedings of the IEEE Conference on Computer Vision and Pattern Recognition (CVPR).

[46] Miikkulainen R., Liang J., Meyerson E., Rawal A., Fink D., Francon O., Raju B., Shahrzad H., Navruzyan A., Duffy N. (2019). Evolving deep neural networks. Artificial Intelligence in the Age of Neural Networks and Brain Computing.

[47] Real E., Moore S., Selle A., Saxena S., Suematsu Y.L., Tan J., Le Q.V., Kurakin A. Large-Scale Evolution of Image Classifiers. Proceedings of the 34th International Conference on Machine Learning.

[48] Shlens J. (2014). A tutorial on principal component analysis. arXiv.

[49] Wang Y., Rong Y., Pan H., Liu K., Hu Y., Wu F., Peng W., Xue X., Chen J. (2020). PCA Based Kernel Initialization for Convolutional Neural Networks. Data Mining and Big Data.

